# An intersectional examination of the relationship between racial/ethnic discrimination and psychotic-like experiences: the role of other psychiatric symptoms

**DOI:** 10.1192/j.eurpsy.2024.1796

**Published:** 2025-01-13

**Authors:** Arielle Ered, Emily Lipner, Kathleen J. O’Brien, Zeeshan M. Huque, Deidre M. Anglin, Lauren M. Ellman

**Affiliations:** 1 Department of Psychiatry, Perelman School of Medicine, University of Pennsylvania, Philadelphia, PA, USA; 2 Department of Psychology and Neuroscience, Temple University, Philadelphia, PA, USA; 3 Department of Psychology, The City College of New York, New York, NY, USA; 4 The Graduate Center, The City University of New York, New York, NY, USA

**Keywords:** discrimination, intersectionality, psychotic-like experiences, gendered racism, multiple mediation

## Abstract

**Background:**

Racial and ethnic experiences of discrimination (EODs) are associated with numerous psychiatric symptoms, including outcomes along the psychosis spectrum; however, less is known about mechanisms by which EODs confer risk for psychotic-like experiences (PLEs; common subthreshold psychotic symptoms). Furthermore, work on gendered racism asserts that the intersection of race and gender impacts the nature of EODs experienced and, in turn, may impact the relationship between EODs and PLEs.

**Aims:**

To utilize an intersectional lens (race and gender) to examine whether psychological correlates of EODs (post-traumatic stress, anxiety, depression, and dissociation) mediate the EOD–PLE relationship.

**Methods:**

Undergraduates at a diverse, semipublic university (*N* = 1,759) completed self-report questionnaires (Experiences of Discrimination Scale, Prodromal Questionnaire, Center for Epidemiologic Studies Depression Scale, State–Trait Anxiety Inventory, Dissociative Experiences Scale, and Post-Traumatic Stress Disorder Checklist – Civilian Version). Analyses stratified the sample by race (non-Hispanic White, Black, and Asian) and examined three multiple mediation models, moderated by gender, examining the pathway from EODs to PLEs, through other psychiatric symptoms.

**Results:**

In the full sample, all psychiatric symptoms significantly mediated the relationship between EODs and PLEs. Only depression varied by gender, such that the indirect effect was only significant in female participants (*β* = 0.09; 95% CI [0.02, 0.16]). Across race-stratified groups, significant mediators varied by both race and gender.

**Conclusions:**

These findings underscore the importance of accounting for intersectionality and multiple psychological symptoms in understanding the EOD–PLE associations, which differ by race and ethnicity as well as gender, and should be considered in clinical treatment of individuals with PLEs and history of EODs.

## Introduction

### Experiences of discrimination and mental health

In the United States (US), mental health disparities between racial and ethnic minorities and White individuals are compounded by several social determinants of health, including experiences of discrimination (EODs), structural racism, police violence, and neighborhood and socioeconomic factors [1]. Psychotic-like experiences (PLEs; subthreshold, attenuated forms of positive psychotic symptoms) in particular are reported more frequently by underrepresented minorities (URMs), including Black and Latinx individuals [2]. While evidence supporting the relationship between EODs and psychosis is robust [3], the mechanisms by which EODs confer risk for PLEs have yet to be fully elucidated. Exploring these mechanisms may be a key to further understanding how to help those who may be at risk for developing psychosis.

The race-based traumatic stress theory suggests that discrimination is experienced as a chronic psychological and physiological stressor among URMs and is associated with negative psychological sequelae [4]. Experiencing discrimination confers risk for symptoms of some of the most common forms of psychopathology, including anxiety, depression, dissociation, and posttraumatic stress [5–7]. In turn, all of these psychological symptoms have been further associated with PLEs [8]. Thus, psychological symptoms may play a critical role in the relationship between EODs and increased PLEs, though no studies have yet examined the indirect effects of psychological symptoms in this relationship. Additionally, it is important to examine the specific symptom pathways through which EODs are associated with PLEs separately by race and ethnicity, as various racial and ethnic groups may be subject to discrimination in different ways (i.e., frequency, severity, and quality) within specific contexts.

Moreover, studies show that the prevalence of psychiatric symptoms often varies by gender and race/ethnicity [9]. Specifically, evidence suggests that Black males show higher rates of anxiety symptoms compared with other males and that Hispanic females show higher rates of anxiety and depression symptoms compared with all other groups [10]. Additionally, Hispanic and Black adolescents are more likely to experience post-traumatic stress disorder (PTSD) symptoms following poly-victimization compared with White adolescents [11]. Further, EODs have been shown to increase risk for suicidal thoughts through the effects of traumatic stress and depressive symptoms, though this relationship was strongest for young women of various racial and ethnic backgrounds [4]. As such, examining these processes in large, nonclinical samples, researchers may disentangle the mechanisms through which PLEs develop across genders and racial/ethnic groups prior to the onset of more severe illness-related factors that may impact symptomatology, such as the use of antipsychotic medications.

### Gendered racism and intersectionality

EODs occur not only based on race and ethnicity but also on a host of other identity-related factors including sexual orientation, disability, socioeconomic status, and, importantly for this work, sex and/or gender [12]. Notably, gender differences have been found in studies of psychiatric symptoms in adolescents, even when racial differences have not been found, with females experiencing significantly higher rates of depression, anxiety, and general emotional distress compared with males, regardless of race [13]. The intersection of race- and gender-based discrimination, or gendered racism, is experienced as individuals hold multiple concurrent identities [14]. Within this framework, it is proposed that racial and gender-specific experiences of distress cannot be viewed separately, but rather intertwine to create a qualitatively different intersecting identity that differentially influences the types of discrimination received [15, 16]. These various intersecting identities may also impact ways that protective factors are accessed, including social support or access to behavioral health care. For example, tailored social support for racial discrimination significantly moderated the relationship between racial discrimination and depression in a sample of Black women [17]. Similarly, social support buffered the impact of racial discrimination on depressive symptoms for Asian Americans during the COVID-19 pandemic [18]. While many studies have examined the relationship between EODs and PLEs, few have accounted for how nuances of intersectionality (both racial and gender identities) further complicate this relationship [1, 19]. Given that gendered racism has been shown to impact psychiatric symptoms commonly associated with PLEs, like depressive symptoms, additional research is needed.

### Current study

The present study surveyed a sample of diverse undergraduates to examine the role of psychiatric symptoms in the relationship between EODs and PLEs. In the overall sample, we hypothesized that symptoms of depression, anxiety, dissociation, and PTSD would mediate the relationship between EODs and PLEs and that gender would impact the symptoms through which EODs and PLEs are related. Next, we examined how intersectional facets of identity (race and gender) impact the relationship between EODs and PLEs via these psychiatric symptoms by repeating the analyses in race-stratified models. As intersectionality theory states that individuals are situated at the intersection of sexism, racism, and other forms of discrimination based on aspects of their identity [12], we do not make specific hypotheses based on group identity, but rather that these processes will differ between groups based on race and gender. We hypothesized that specific racial and ethnic group and gender, together, would impact the symptoms through which EODs are associated with PLEs.

## Methods

### Participants

Participants included 1,759 undergraduates from a large, urban university who were recruited through the university’s online research participant system. Thus, this sample is nonclinical in nature, but individuals may have been engaged in or seeking out mental health treatment. Participants completed questionnaires on laboratory computers with staff available to answer questions.

### Ethics statement

The authors assert that all procedures contributing to this work comply with the ethical standards of the relevant national and institutional committees on human experimentation and with the Helsinki Declaration of 1975, as revised in 2008. All procedures involving human subjects were approved by Temple University Institutional Review Board Protocol #13359, *Psychotic-Like Experiences in a Non-Clinical Young Adult Student Population.* Written informed consent was obtained from all participants prior to study participation.

### Measures

#### Demographics

Demographic characteristics were self-reported by participants. Race was reported using National Institute of Health racial categories, and included “American Indian/Alaska Native,” “Asian,” “Native Hawaiian/Pacific Islander,” “Black,” “White,” and “Biracial.” Ethnicity was categorized as “Hispanic/Latino” or “non-Hispanic/Latino.” Gender identity (notably, not sex assigned at birth) was categorized as “male” or “female.” Gender was selected due to data availability, as the original study assessed gender identity rather than sex. Additional sociodemographic variables, including urbanicity of upbringing and immigrant status, were also self-reported.

#### Discrimination

EODs were measured using the Experiences of Discrimination Scale [20]. This 11-item self-report scale measures perceived discrimination due to race, ethnicity, or skin color in nine different situations (e.g., school, work, public, and legal) over the individual’s lifetime. This scale has been validated for use with White, Black, and Latinx Americans [20]. The total frequency of EODs across contexts was the independent variable in all models examined continuously.

#### Psychiatric symptoms

PLEs were measured using the positive subscale of the Prodromal Questionnaire (PQ) [21]. The PQ is a 92-item self-report questionnaire that assesses the presence of positive, negative, disorganized, and general symptoms of psychosis experienced in the past month. Additionally, this scale assesses whether these experiences are distressing. Participants are asked to report on their experiences in the absence of substance and medication use. This scale has demonstrated good concurrent validity with interview-rated clinical high-risk diagnoses when individuals endorse eight or more positive items as distressing [21, 22]. The PQ positive subscale (sum of 45 items) was the dependent variable in all models and was examined continuously.

Depression, anxiety, dissociative, and PTSD symptoms were examined as continuous mediators and were measured using four separate self-report measures. Symptoms of depression were measured using the brief version of the Center of Epidemiologic Studies Depression Scale (CES-D) [23, 24]. The brief CES-D is a 10-item self-report measure that assesses the frequency of cognitive, somatic, and interpersonal symptoms of depression in the past week. The CES-D was developed for a community sample and has demonstrated reliability and validity across racial/ethnic samples [23, 25]. Scores may range from 0–30; summed score was examined for the present analyses.

Generalized anxiety symptoms were measured using the State–Trait Anxiety Inventory, Trait Form, Anxiety Subscale (STAI) [26]. Factor analysis of the full Trait Form demonstrates a two-factor structure for anxiety and depression; depression items were removed for the present study to minimize overlap with CES-D symptoms and target anxiety specifically [27]. This 7-item scale (score range 7–28) asks participant to report the frequency at which they experience various symptoms of anxiety. Summed scores were examined continuously for the present analyses.

Dissociative experiences were measured using the Dissociative Experiences Scale (DES) [28]. The DES is a 28-item, self-report measure that assesses the extent to which individuals experience a range of dissociative experiences (i.e., derealization and depersonalization) on a 0–100 scale, indicating the percentage of time the symptom is experienced. The DES is a widely used, highly reliable, and valid measure of dissociative symptoms and is sensitive to the identification of these symptoms transdiagnostically and in nonpsychiatric samples [29, 30]. The mean frequency score was used for the present analyses.

PTSD symptoms were measured using the Post-Traumatic Stress Disorder Checklist – Civilian Version (PCL-C), a measure generalized from the PCL-Military Version for use in broader populations [31]. This 17-item self-report measure specifically assesses DSM-IV symptoms of PTSD in the past month. Of note, no item of the PCL-C explicitly assesses for dissociative experiences. This scale demonstrates strong reliability and validity in clinical, nonclinical, and undergraduate samples [31, 32]. Summed scores (possible range 17–85) were used for the present analyses.

#### Data analysis

All analyses were conducted in R (v 3.6.2). Pearson correlations were calculated to examine all bivariate relationships between the Independent Variable (IV), Dependent Variable (DV), mediators, and continuous demographic variables (i.e., age) relevant to the present study. Demographic variables significantly associated with both the IV and DV (i.e., age) were included in the main analyses as covariates.

The lavaan package in R was used to estimate all models [33]. First, a moderated multiple mediation model examined the relationship between EODs and PLEs via four mediators (depressive, anxiety, dissociative, and PTSD symptoms), and the impact of gender on these relationships, for the full analytic sample. All mediators were standardized to allow for comparison between symptoms scales. Within this model, we also calculated indirect effects for males and females separately regardless of significant interaction, as our aim was to examine gendered racism. Mediation effects were tested using a bootstrap estimation approach with 5,000 samples. Covariances between the four mediators were included in all models. Significant indirect effects were indicated when the 95% confidence interval did not include zero [34].

The sample was then stratified into three racial subgroups sufficiently powered for the present analyses (non-Hispanic White [NHW], Black, and Asian) to examine these relationships. We examined individuals who identified as NHW due to the relatively low incidence of Hispanic-identifying individuals in our sample (*n* = 62) and because of the unique impact of Latinx identity in the US on EODs [35]. While we anticipate that NHW individuals will have experienced fewer incidences of racial discrimination, it is important to examine this group as it may include individuals of Middle Eastern and North African descent who often have to report their race as White despite experiencing discrimination based on skin color, and NHW individuals report experiencing racial discrimination in other studies [36], although they are not structurally impacted by racism in the same way as minoritized racial groups. Moderated multiple mediation was repeated in each subgroup utilizing the specifications described above.

## Results

### Sample overview

Of the 1,759 participants included in this sample, 950 identified as NHW, 325 identified as Black, and 270 identified as Asian. Demographic characteristics of the present sample are displayed in Table 1. Correlations between all study variables are available in Supplementary Table 1. As age was significantly associated with both EODs and PLEs, all direct and indirect effects were estimated controlling for age.

**Table 1.** Descriptive statistics for the full analytic sample and by racial and ethnic subgroup

	Full sample	NHW	Black	Asian	*F* or *χ* ^2^
	(*n* = 1,759)	(*n* = 950; 54%)	(*n* = 325; 18%)	(*n* = 270; 15%)	
Gender (male), *n* (%)	432 (25%)	243 (25%)	64 (20%)	163 (60%)	8.52*
Age (years), *M* (SD) [range]	20.13 (2.42) [18–34]	20.05 (2.33) [18–34]	20.74 (3.15) [18–34]	19.93 (1.79) [18–30]	0.60
Immigrant, *n* (%)	216 (12%)	49 (5%)	40 (12%)	177 (66%)	139.08^**^
Urbanicity, *n* (%)					230.44^**^
Rural	199 (11%)	142 (15%)	27 (8%)	45 (9%)	
Suburban	1,078 (61%)	679 (71%)	120 (37%)	293 (56%)	
Urban	450 (26%)	124 (13%)	170 (52%)	185 (35%)	
Other	32 (2%)	14 (2%)	8 (3%)	4 (1%)	
EOD frequency, *M* (SD) [range]	2.67 (4.42) [0–35]	1.03 (2.33) [0–20]	5.80 (6.21) [0–33.5]	3.98 (4.48) [0–20]	219.80^**^
PQ positive, *M* (SD) [range]	9.82 (7.59) [0–44]	9.49 (7.67) [0–42]	9.74 (7.35) [0–44]	10.58 (7.45) [0–37]	4.05*
CES-D total, *M* (SD) [range]	7.97 (5.29) [0–27]	7.83 (5.4) [0–26]	8.28 (4.82) [0–23]	7.94 (5.32) [0–26]	0.64
STAI total, *M* (SD) [range]	12.68 (4.87) [7–28]	12.66 (4.86) [7–28]	12.29 (4.48) [7–26]	13.30 (5.15) [7–28]	2.51
DES mean, *M* (SD) [range]	12.96 (12.38) [0–77.86]	11.70 (11.05) [0–77.86]	15.65 (13.85) [0–75.36]	13.65 (13.81) [0–77.14]	11.22^**^
PCL-C total, *M* (SD) [range]	31.56 (12.89) [17–85]	31.69 (12.92) [17–84]	29.98 (11.85) [17–68]	32.08 (12.98) [17–74]	0.03

Abbreviations: CES-D, Center for Epidemiological Studies Depression Scale; DES, Dissociative Experiences Scale; EOD, Experiences of Discrimination Scale; *M*, mean; NHW, non-Hispanic White; PCL-C, Post-Traumatic Stress Disorder Checklist – Civilian Version; PQ, Prodromal Questionnaire; SD, standard deviation; STAI, State Trait Anxiety Index, Trait Form, Anxiety Subscale.

* *p* < 0.05.

** *p* < 0.001.

### Multiple mediation models

#### Full sample

The full sample consisted of all individuals in the sample, including individuals from racial and ethnic backgrounds that were not powered to test individually. All direct effects were significant, such that EODs were associated with increased psychiatric symptoms and all four symptoms were associated with increased PLEs (see Supplementary Figure 1). Further, there was a significant direct effect of EODs on PLEs (see the Supplementary Material for details). As can be seen in Table 2, there was a significant indirect effect of dissociation and PTSD symptoms for both males and females in the full sample. There was a significant indirect effect of depression and anxiety symptoms for females, but not for males. A sensitivity analysis was conducted including only individuals within the Black, Asian, and NHW subsamples, excluding individuals from groups for which we were not powered to test individually (referred to as the “combined sample”). The results in the combined sample were consistent with the results in the full sample (see Supplementary Figure 2 and Supplementary Table 2).

**Table 2.** Summary of indirect effects in all moderated multiple mediation models

	Full sample		NHW		Black		Asian	
	*β*	95% CI	*β*	95% CI	*β*	95% CI	*β*	95% CI
*All genders*								
Depression	**0.08**	**[0.02, 0.15]**	0.08	[0.00, 0.15]	0.06	[−0.05, 0.16]	0.19	[−0.15, 0.53]
Anxiety	**0.19**	**[0.10, 0.28]**	**0.16**	**[0.05, 0.27]**	0.11	[−0.10, 0.32]	**0.43**	**[0.05, 0.81]**
Dissociation	**0.31**	**[0.21, 0.41]**	**0.45**	**[0.28. 0.61]**	0.07	[−0.03, 0.25]	0.13	[−0.04, 0.31]
PTSD	**0.26**	**[0.15, 0.36]**	**0.25**	**[0.11, 0.38]**	**0.45**	**[0.15, 0.75]**	**0.46**	**[0.11. 0.80]**
*Males*								
Depression	0.07	[−0.01, 0.14]	0.06	[−0.03, 0.14]	0.05	[−0.09, 0.20]	0.21	[−0.19, 0.61]
Anxiety	0.13	[−0.02, 0.28]	0.14	[−0.01, 0.29]	−0.10	[−0.50, 0.31]	**0.59**	**[0.03, 1.15]**
Dissociation	**0.31**	**[0.15, 0.48]**	**0.54**	**[0.31, 0.78]**	0.06	[−0.19, 0.31]	0.21	[−0.10, 0.52]
PTSD	**0.25**	**[0.07, 0.43]**	**0.30**	**[0.11, 0.49]**	0.30	[−0.24, 0.83]	**0.54**	**[0.01, 1.06]**
*Females*								
Depression	**0.09**	**[0.02. 0.16]**	0.10	[0.00, 0.20]	0.06	[−0.06, 0.18]	0.18	[−0.15, 0.50]
Anxiety	**0.18**	**[0.10, 0.31]**	**0.18**	**[0.04, 0.32]**	0.19	[−0.06, 0.43]	**0.37**	**[0.02, 0.72]**
Dissociation	**0.28**	**[0.20, 0.42]**	**0.36**	**[0.16, 0.56]**	0.13	[−0.03, 0.29]	0.11	[−0.08, 0.30]
PTSD	**0.25**	**[0.14, 0.38]**	**0.22**	**[0.06, 0.38]**	**0.53**	**[0.18, 0.87]**	**0.42**	**[0.06, 0.78]**

Abbreviations: NHW, non-Hispanic White; PTSD, post-traumatic stress disorder.

Bolded entries indicate significance, do not contain 0.

#### Black subsample

Among Black participants, there were no significant interactions between EODs and gender predicting psychiatric symptoms. As can be seen in Supplementary Figure 3, EODs were directly associated with increased depressive and PTSD symptoms, but not with anxiety or dissociative symptoms. There were also significant direct effects of anxiety, dissociation, and PTSD symptoms on PLEs, such that higher symptom scores were associated with higher PLEs. Among all Black participants, there was a significant indirect effect of PTSD symptoms on the association between EODs and PLEs, but not depression, anxiety, or dissociation symptoms (see Table 2). Separately, the indirect effect of PTSD symptoms existed only for females, though it should be noted that Black males represented the smallest group in the sample (*n* = 64), potentially leading to lack of power.

#### Asian subsample

Among Asian participants, there were no significant interactions between EODs and gender predicting psychiatric symptoms. There were significant direct effects of EODs on depression, anxiety, and PTSD symptoms, such that higher EODs were associated with higher symptom scores (see Supplementary Figure 4). Further, higher anxiety, dissociative, and PTSD symptoms were directly associated with higher PLEs. Among all Asian participants, there was a significant indirect effect of anxiety and PTSD symptoms, but not depression or dissociation symptoms, which remained consistent for Asian males and females separately.

#### Non-Hispanic White subsample

Among NHW participants, there were no significant interactions between EODs and gender predicting psychiatric symptoms. As can be seen in Supplementary Figure 5, all direct effects were significant, such that higher number of EODs were associated with higher scores on all four symptom measures. Further, all symptom measures were associated with a greater number of PLEs. As expected, EODs were also associated with increased PLEs. For all NHW individuals, there was a significant indirect effect of anxiety, dissociative, and PTSD symptoms on the association between EODs and PLEs, which remained consisted for NHW females (see Table 2). For NHW males, there was a significant indirect effect of dissociative and PTSD symptoms, but not depression or anxiety symptoms.

## Discussion

In sum, this study presents evidence that depression, dissociation, anxiety, and PTSD symptoms mediate the relationship between experiences of racial discrimination and PLEs and, while there is an overlap in mediators between groups, these relationships vary both by race and gender. In the full sample, all psychiatric symptoms significantly mediated the EOD–PLE relationship; however, in the race-stratified subgroups, this was not the case. Our findings underscore the importance of probing the impacts of intersectional identities on psychological response to discrimination-based stress.

In our sample, the highest frequency of EODs was reported by Black participants. In this subgroup, only PTSD symptoms significantly mediated the relationship between EODs and PLEs. Discrimination can be a stressful and potentially traumatic experience, and a salient one for Black communities in the US [4]. Racism at a systemic and structural level can additionally increase the risk of exposure to traumatic life events [37]. There are biological and psychological phenotypes that overlap between Black American samples faced with interpersonal and systemic racism and samples of individuals with a PTSD diagnosis, including decreased basal cortisol levels [38, 39] and chronic systemic inflammation [40, 41]. As such, it is vital for clinicians and researchers to assess the incidence of PTSD symptoms, even in the absence of PTSD diagnosis, in Black individuals.

We did not identify any symptom x gender interactions among Black participants; however, only 20% of Black individuals in this study were male, despite Black men being well-represented in the university from which the study population was drawn, potentially limiting our ability to examine these effects in Black males. It is possible that there were also race x gender effects in terms of willingness and interest in participating in a research study [42]. It is also important to note that PTSD symptoms, distinct from dissociative experiences, significantly mediated these relationships, even as Black participants reported the highest endorsement of dissociative experiences in this sample, suggesting that other PTSD-related symptoms (e.g., hypervigilance) may contribute to increased PLEs.

Anxiety and PTSD symptoms significantly mediated the EOD–PLE relationship in Asian participants, and was consistent between males, females, and the combined gender sample. Interestingly, our sample of Asian individuals had a relatively large proportion of immigrants (66%), which may impact potentially discriminatory treatment of these individuals in the US and the psychological and cognitive mechanisms through which PLEs develop following EODs.

Our findings demonstrate that depression, anxiety, dissociation, and PTSD symptoms all significantly mediated the relationship between EODs and PLEs in the NHW subgroup. These indirect effects vary by gender, such that the indirect effects of depression and anxiety are significant in only female NHW participants. There may be numerous reasons that NHW female participants might perceive EODs and how they impact their mental health differently from their male counterparts. It is also possible that NHW individuals report experiencing discrimination on the basis of their skin tone, though a recent study found that self-reported variations in skin tone is associated with perceptions of how others might perceive one’s race or ethnicity but not daily EODs in White individuals [43]. Additionally, studies have found that NHW men are more likely to both anticipate and perceive themselves as experiencing reverse racism (or anti-majority racial discrimination) [14]. It is also possible that the NHW females answered about EODs related to gender, rather than skin color, as the measure instructs.

It is important to note that this study utilized NIH categories for racial groups. Although these categories are the field standard for collecting and reporting racial identity, these groups are large “bins” that may mask within-group heterogeneity relevant to EODs in the US. For example, both individuals of European and Middle Eastern and North African ancestry are categorized within the “White” category, despite variability in experiences of racial and ethnic trauma and discrimination within the US [44]. Furthermore, within our sample, there is heterogeneity within each racial subgroup of both immigrant status and urbanicity, two factors that have been shown to be significantly associated with psychosis risk in the US [45]. While probing these groups further would limit power in the present analyses, it is important to acknowledge that these racial categories are quite nuanced and researchers should consider alternative statistical techniques that may be able to comprehensively examine intersectional facets of identity [46].

The present study placed an emphasis on participants’ subjective experiences of their psychiatric symptoms; however, future studies should also assess the cognitive and neurobiological underpinnings of psychiatric symptoms in the relationship between EODs and PLEs, which may also be impacted by race and gender differences. For example, negative beliefs and schemas have been implicated in the relationship between traumatic exposures and psychosis [47, 48], as well as between EODs and psychotic symptoms [3]. EODs have also been associated with heightened stress responses and dysregulated hypothalamus–pituitary–adrenal axis functioning, which may be a transdiagnostic risk factor for psychiatric outcomes [49]. Biomarkers of the stress response (e.g., inflammatory markers and cortisol) and HPA-axis functioning are also known to vary by race and gender [50].

### Strengths and limitations

Our large sample of non-help-seeking individuals with a range of psychiatric symptoms is a significant strength of the present study. The university is housed within a large, urban city and captures more ethnic and racial diversity than the typical college sample and is thus more generalizable to other community samples. The nature of the nonclinical undergraduate sample may have also helped to capture a wider range of PLEs in our sample than if participants were seeking out this research due to a subjective history of bizarre or unusual psychiatric experiences.

Limitations should also be noted and addressed in future research. First, our EOD measure only examined lifetime EODs based on race, ethnicity, and skin color. Measures that consider EODs based on intersectional aspects of identity (race and ethnicity, gender, sexual orientation, disability status, age, etc.) at varying frequencies or within specific, relevant contexts (e.g., healthcare-specific discrimination) should be used to best capture a range of EODs. For example, the Perceived Discrimination Scale, which captures both lifetime and everyday EODs, examines discrimination based on several identity factors within several contexts [51] and has been utilized in other population-based studies within the US. Due to the average age of the sample in the present study, it is also possible that many participants had not yet been exposed to opportunities for discrimination in certain contexts assessed on the EOD scale (e.g., applying for credit, bank loans, or mortgages), although our sample includes “nontraditional” or older students up to 35-year-olds. Nonetheless, these are experiences that they may have observed within their own family, having a direct impact on their life experience to date. There are possible cohort effects as this sample was collected prior to 2016. Sociopolitical events over the last decade have influenced the prevalence of racial discrimination and public awareness of its prominence within the US [52].

Additionally, we utilized self-report measures exclusively in this study. Future studies may choose to include interview-based measures of PLEs to validate these findings. We also examined these models using cross-sectional data. Examining these experiences with temporal precedence, to examine the course of psychiatric sequelae from discrimination to PLEs, would strengthen the meaning of these findings. Finally, we report here on binary gender identity (male/female), failing to capture the full gender identity spectrum and sex assigned at birth. Future studies should collect both gender identity and sex assigned at birth, with options available for diverse identities (e.g., nonbinary, gender nonconforming, and intersex), to assess intersectional facets of identity more comprehensively and accurately.

### Implications

These findings provide insight into targets for therapeutic intervention for individuals depending on their racial and gender identity. The differential symptom pathways emphasize that while ethnoracial discrimination is unfortunately present across groups, these experiences are different based on intersectional aspects of identity *and* the psychiatric symptoms that arise following these experiences differ as well. Clinicians should be aware of the role of EODs and other psychiatric symptoms (e.g., anxiety, depression, and trauma) in the context of PLEs and should assess individuals for clinically relevant symptoms that may require therapeutic intervention (e.g., cognitive processing therapy or prolonged exposure therapy for PTSD), which may in turn improve PLEs. They also underscore and reaffirm the importance of discussing culture and identity in the therapeutic space to increase belongingness and trust, which may be particularly important when working with clients with marginalized racial and gender identities, and with often stigmatized mental health symptoms like PLEs.

These findings also specifically provide implications for the impact of race and gender on psychiatric symptoms in early adulthood, as the present study exclusively examined an undergraduate sample. Early adulthood is a period of increased risk for the emergence of various psychiatric disorders, including psychotic disorders, and may also be a time when individuals are having their first contact with behavioral health systems. Given the significant stigma associated with psychosis symptoms in particular, and the increased risk of PLEs following discriminatory experiences, clinicians should address these issues directly in the therapeutic process and integrate interventions that target internal and external sources of stigma [53]. Intersectional considerations in the relationship between EODs and PLEs in other aged samples, for example, in an older adult sample, would heed additional considerations and likely consider other important psychiatric symptoms like cognitive functioning [54]. As such, our study further clarifies the trajectory from discrimination to PLEs in a unique period of development during which the emergence of psychosis spectrum symptoms is most common.

Results also emphasize the need to better assess clients’ attitudes toward their contextual stressors, symptoms, and mental health care [55]. Clinicians may increase awareness of how national and local events may impact prevalence of EODs in the groups they work with. For example, political rhetoric during election season has been associated with increases in hate crimes and employment discrimination [56, 57] and rises in anti-Asian discrimination during the COVID-19 pandemic were further associated with increases in depression for impacted individuals [18]. Discrimination on the basis of the intersection of race and gender may even be impacting help-seeking behavior prior to the onset of intervention and inform the stage at which an individual first accesses care [58]. Conversely, differences in mental health stigma in specific gender and racial subgroups may impact care. Beyond the individual level, these findings further evidence the need to address issues of structural racism and minimize incidences of discrimination in the community, given its deleterious and transdiagnostic impact on psychological well-being [1]. We must continue to strive to incorporate thoughtful examinations of intersectional identity into research, expanding also upon the facets of identity that are considered.

## Supporting information

Ered et al. supplementary material 1 Ered et al. supplementary material

Ered et al. supplementary material 2 Ered et al. supplementary material

## Data Availability

The data that support the findings of this study are available from the corresponding author, L.M.E., upon reasonable request.

## Supplementary material

The supplementary material for this article can be found at http://doi.org/10.1192/j.eurpsy.2024.1796.
