# Supplementary material for: An intersectional examination of the relationship between racial/ethnic discrimination and psychotic-like experiences: the role of other psychiatric symptoms
Source: Eur Psychiatry. 2025 Jan 13;68(1):e6. doi: 10.1192/j.eurpsy.2024.1796 (PMC11795428; doi:10.1192/j.eurpsy.2024.1796)
Supplement: Ered et al. supplementary material 1 — Ered et al. supplementary material [file S0924933824017966sup001.pdf]

Supplemental Figure 1. Multiple moderated mediation model in the full analytic sample.

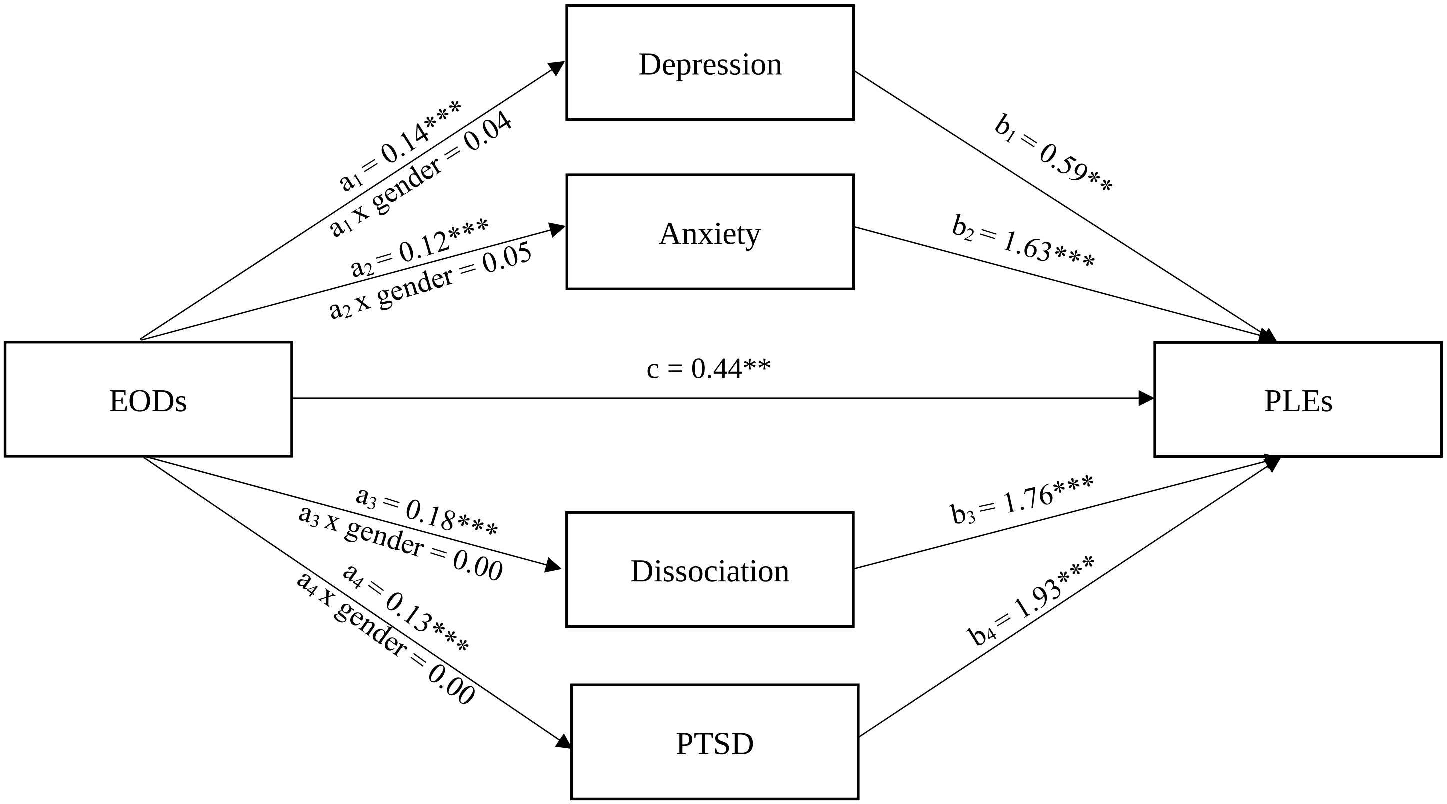

Notes. * p > 0.05, ** p > 0.01, *** p > 0.001; EODs= experiences of discrimination, PLEs= psychotic-like experiences, PTSD= post-traumatic stress disorder

Supplemental Figure 2. Multiple moderated mediation model in the combined analytic sample.

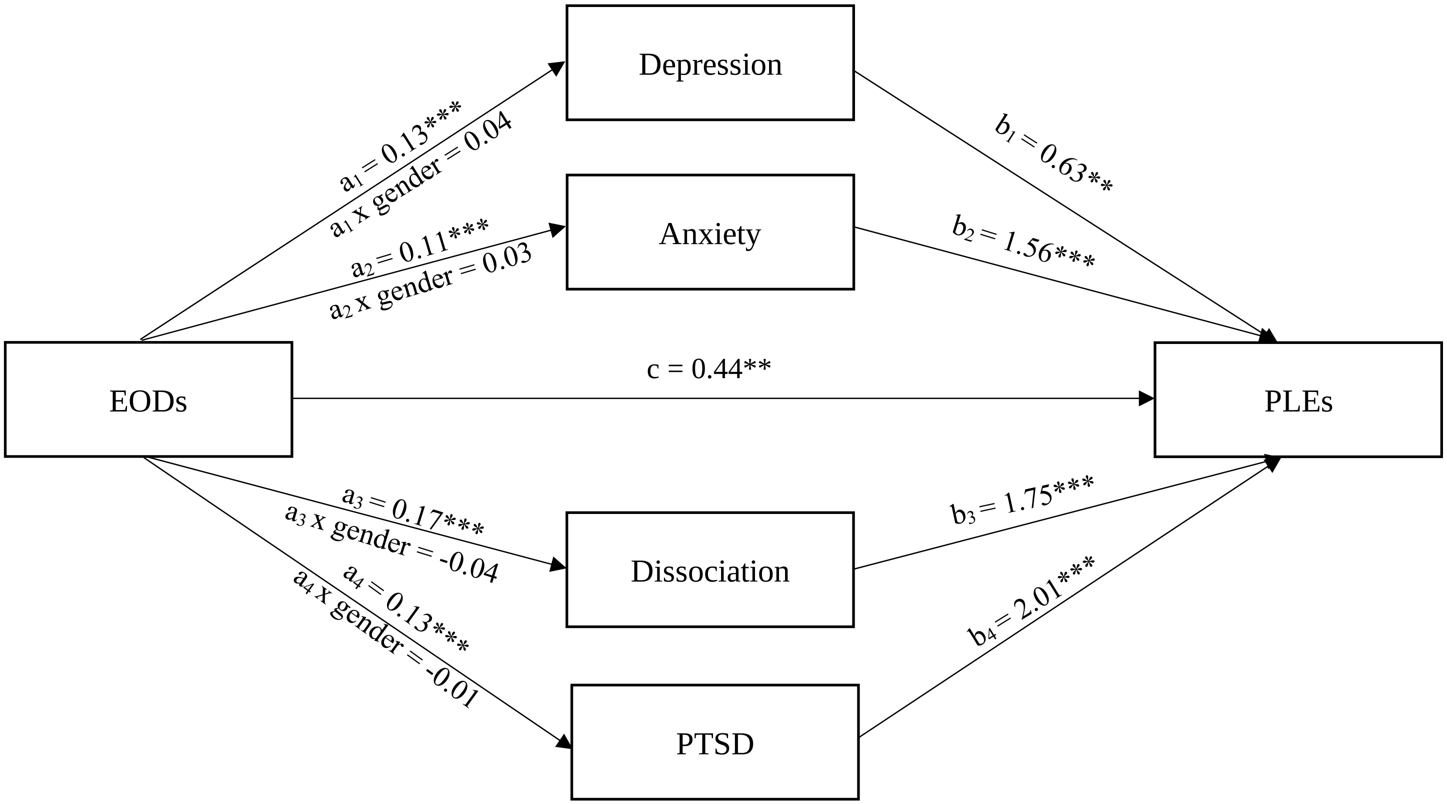

Notes. * p > 0.05, ** p > 0.01, *** p > 0.001; EODs= experiences of discrimination, PLEs= psychotic-like experiences, PTSD= post-traumatic stress disorder

Supplemental Figure 3. Multiple moderated mediation model in the Black subsample.

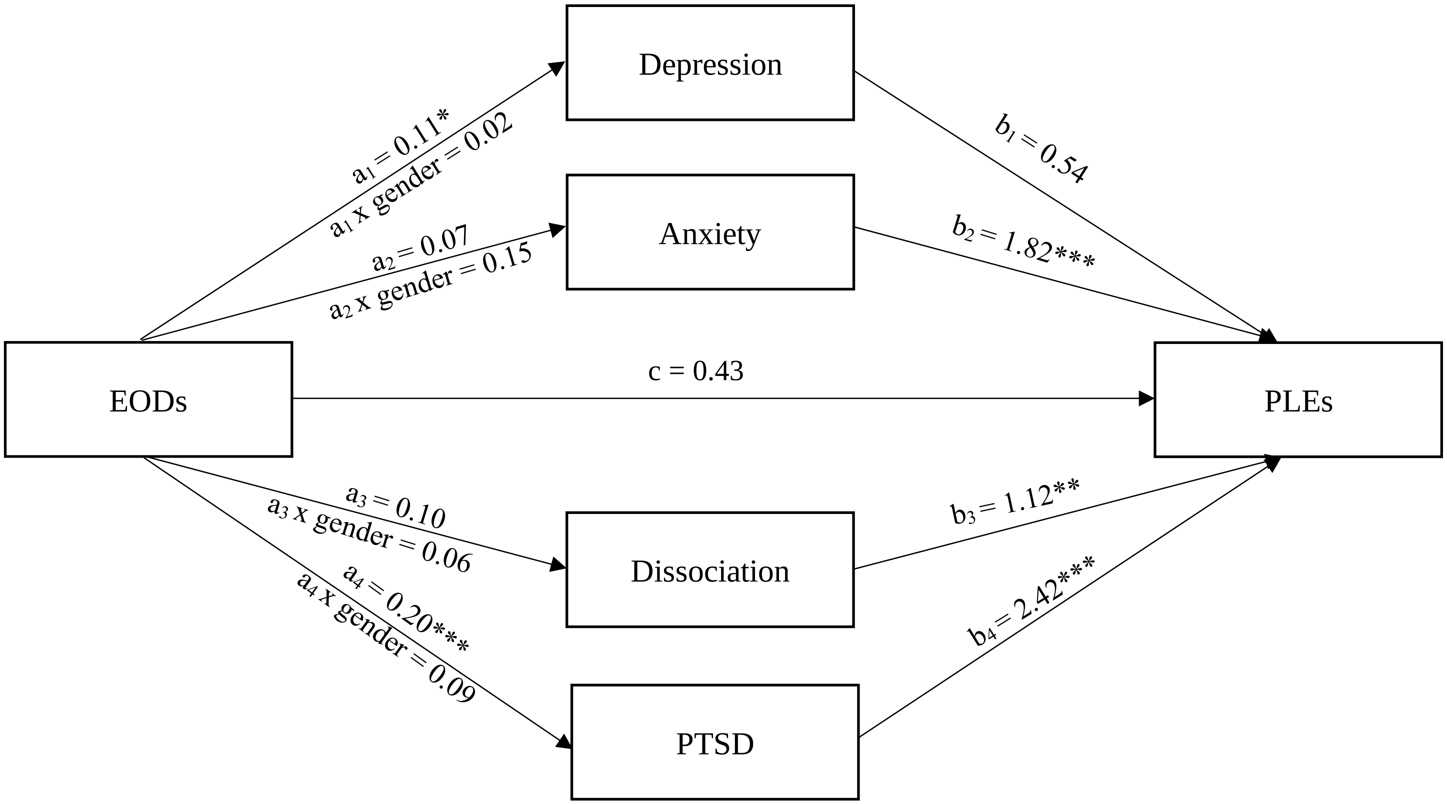

Notes: * p > 0.05, ** p > 0.01, *** p > 0.001; EODs= experiences of discrimination, PLEs= psychotic-like experiences, PTSD= post-traumatic stress disorder

Supplemental Figure 4. Multiple moderated mediation model in the Asian subsample.

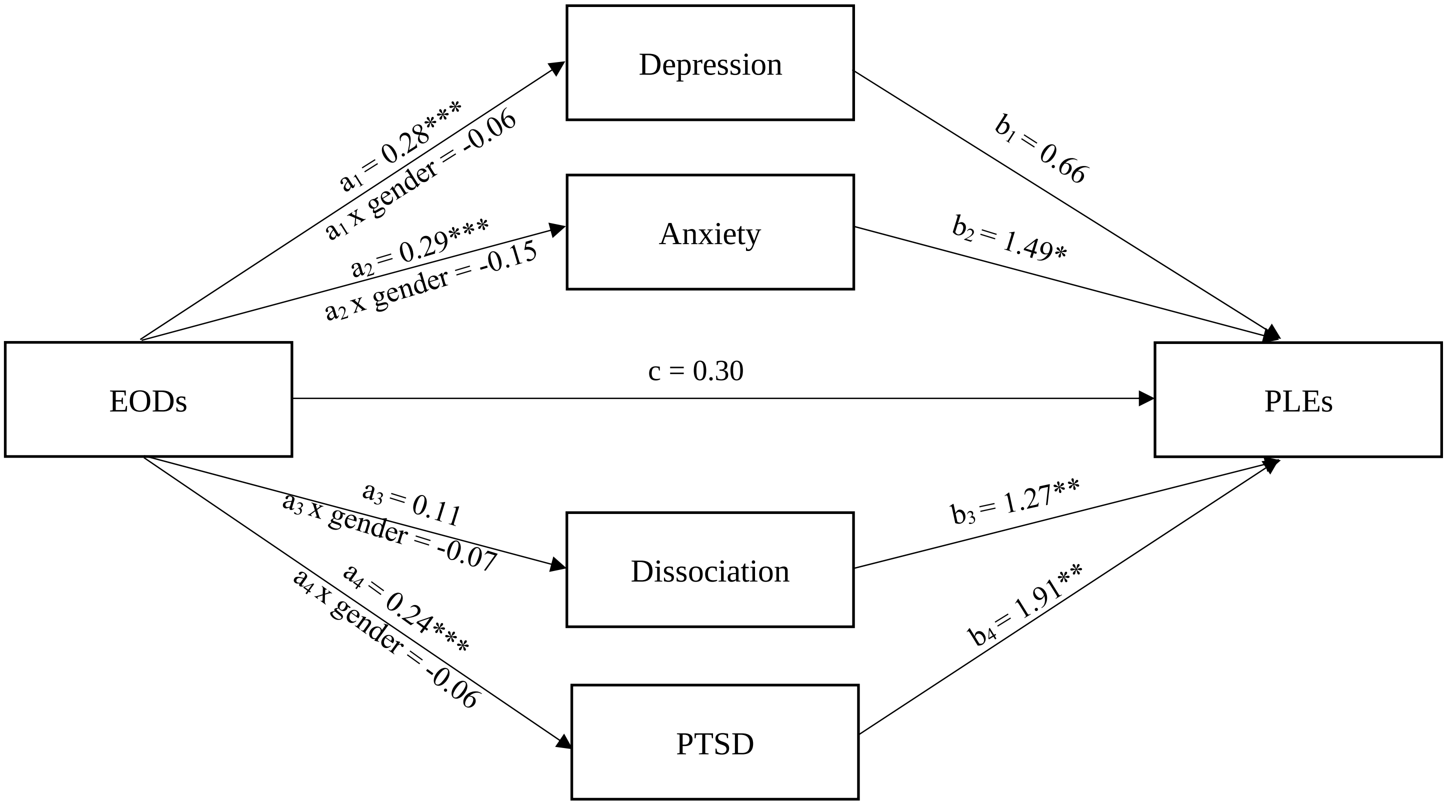

Notes: * p > 0.05, ** p > 0.01, *** p > 0.001; EODs= experiences of discrimination, PLEs= psychotic-like experiences, PTSD= post-traumatic stress disorder

Supplemental Figure 5. Multiple moderated mediation model in the Non-Hispanic White subsample.

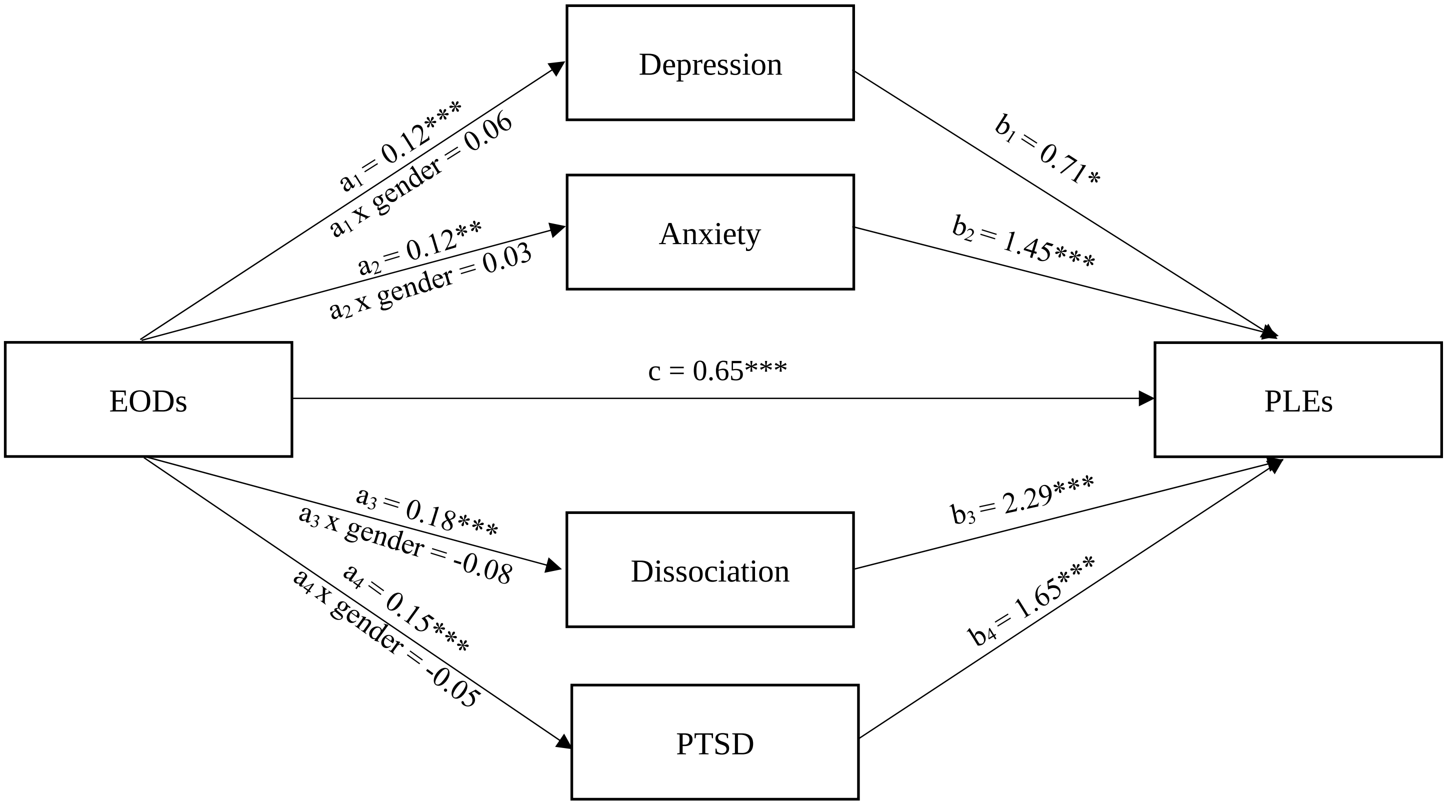

Notes: * p > 0.05, ** p > 0.01, *** p > 0.001; EODs= experiences of discrimination, PLEs= psychotic-like experiences, PTSD= post-traumatic stress disorder
