# Supplementary material for: An intersectional examination of the relationship between racial/ethnic discrimination and psychotic-like experiences: the role of other psychiatric symptoms
Source: Eur Psychiatry. 2025 Jan 13;68(1):e6. doi: 10.1192/j.eurpsy.2024.1796 (PMC11795428; doi:10.1192/j.eurpsy.2024.1796)
Supplement: Ered et al. supplementary material 2 — Ered et al. supplementary material [file S0924933824017966sup002.docx]

| Supplemental Table 1. Correlation matrix for full sample (n= 1759) | | | | | | | |
| --- | --- | --- | --- | --- | --- | --- | --- |
|  | PQ | CES-D | STAI | DES | PCL-C | EOD | Age |
| PQ Positive | - |  |  |  |  |  |  |
| CES-D | .51** | - |  |  |  |  |  |
| STAI | .56** | .71** | - |  |  |  |  |
| DES | .49** | .40** | .42** | - |  |  |  |
| PCL-C | .58** | .72** | .70** | .48** | - |  |  |
| EOD | .16** | .13** | .10** | .17** | .12** | - |  |
| Age | -.07** | -.02 | -.08** | -.03 | -.05 | .13** | - |
| Note: PQ= Prodromal Questionnaire; CES-D=Center for Epidemiological Studies-Depression Scale; STAI=State Trait Anxiety Index, Trait Form, Anxiety Subscale; DES=Dissociative Experiences Scale; PCL-C= Post-traumatic Stress Disorder Checklist- Civilian Version; EOD= Experiences of Discrimination Scale; | | | | | | | |

| Supplemental Table 2. Indirect effects for combined sample (NHW, Black, Asian; n= 1545) | | |
| --- | --- | --- |
|  | β | 95% CI |
| All Genders | | |
| Depression | **0.08** | **[0.02, 0.15]** |
| Anxiety | **0.17** | **[0.08, 0.26]** |
| Dissociation | **0.30** | **[0.19, 0.40]** |
| PTSD | **0.26** | **[0.14, 0.37]** |
| Males | | |
| Depression | 0.07 | [-0.01, 0.14] |
| Anxiety | 0.14 | [-0.01, 0.29] |
| Dissociation | **0.34** | **[0.17, 0.52]** |
| PTSD | **0.27** | **[0.07, 0.46]** |
| Females | | |
| Depression | **0.09** | **[0.02. 0.16]** |
| Anxiety | **0.18** | **[0.08, 0.29]** |
| Dissociation | **0.28** | **[0.16, 0.39]** |
| PTSD | **0.25** | **[0.12, 0.39]** |
| Note. PTSD= post-traumatic stress disorder | | |
